# ‘Inert’ ingredients are understudied, potentially dangerous to bees and deserve more research attention

**DOI:** 10.1098/rspb.2021.2353

**Published:** 2022-03-09

**Authors:** Edward A. Straw, Linzi J. Thompson, Ellouise Leadbeater, Mark J. F. Brown

**Affiliations:** ^1^ Centre for Ecology, Evolution and Behaviour, Department of Biological Sciences, School of Life Sciences and the Environment, Royal Holloway University of London, Egham, Surrey TW20 0EX, UK; ^2^ Department of Agriculture and Food Science, University College Dublin, Belfield, Dublin, Ireland

**Keywords:** inert ingredients, adjuvant, co-formulant, bees, systematic review, pesticides

## Abstract

Agrochemical formulations are composed of two broad groups of chemicals: active ingredients, which confer pest control action, and ‘inert’ ingredients, which facilitate the action of the active ingredient. Most research into the effects of agrochemicals focusses on the effects of active ingredients. This reflects the assumption that ‘inert’ ingredients are non-toxic. A review of relevant research shows that for bees, this assumption is without empirical foundation. After conducting a systematic literature search, we found just 19 studies that tested the effects of ‘inert’ ingredients on bee health. In these studies, ‘inert’ ingredients were found to cause mortality in bees through multiple exposure routes, act synergistically with other stressors and cause colony level effects. This lack of research is compounded by a lack of diversity in study organism used. We argue that ‘inert’ ingredients have distinct, and poorly understood, ecological persistency profiles and toxicities, making research into their individual effects necessary. We highlight the lack of mitigation in place to protect bees from ‘inert’ ingredients and argue that research efforts should be redistributed to address the knowledge gap identified here. If so-called ‘inert’ ingredients are, in fact, detrimental to bee health, their potential role in widespread bee declines needs urgent assessment.

## 1. Introduction

Ecosystem services provided by pollinators contribute $235–577 billion to the global economy each year, with bees providing the majority of pollination [1]. However, declines in bees have been identified, with, for example, 37% of European bee species with known population trends being in decline [2]. This poses a significant threat to the economic value bees provide [1]. Numerous factors may contribute, but one that has been repeatedly implicated based on correlational, experimental and modelling data at a range of scales is the widespread use of pesticides [3–6]. However, pesticides are not applied alone, but are used within complex formulations. Each formulation includes both the active ingredient itself, and co-formulants that facilitate the action of the active ingredient [7]. When applied to crops, such formulations are often further accompanied by separate products added to the tank mixture called adjuvants that complement the action of the pesticide. Both co-formulants and adjuvants play a range of roles, including as surfactants that help active ingredients penetrate leaves, emulsifiers that help products stay thoroughly mixed, and solvents that help to dissolve the active ingredient [7]. These substances are referred to as ‘inert’ ingredients, because they are not intended to have direct pest control action.

There are no comprehensive figures for global ‘inert’ use, as California is the only regulatory zone to accurately record their application [8,9], but they are known to be heavily used globally. According to the United States (US) federal Environmental Protection Agency, there are around 4000 ‘inert’ ingredients in use in the US [10]. No equivalent data are available for the European Union (EU) as a whole, but there are, for example, 294 adjuvant products and 2892 pesticide products registered for use in the UK [11,12]. As almost all active ingredients are applied as part of formulations, all formulations contain co-formulants, and formulations are commonly sprayed in a tank mix containing an adjuvant product, we can surmise that the quantity of ‘inert’ ingredient application is commensurate to, or likely even exceeds, that of active ingredients. Further, no mitigation measures are attached to adjuvants, meaning they can often be sprayed onto crops while bees forage on them. Co-formulants typically only have mitigation measures carried over from the active ingredient, not measures tailored to their specific toxicity. Thus, the exposure of bees to them, though unquantified, is likely to be considerable.

While regulatory bodies require active ingredients to undergo a suite of toxicity testing on bees (e.g. [13–16]), no parallel testing is required for individual ‘inert’ ingredients [13,14], despite evidence of potential toxicity [17,18]. Instead, in the EU, there is toxicity testing of a single commercial product per active ingredient, called the ‘representative formulation’ [19], while in the US, only the toxicity of the active ingredient is considered [13,20]. In the EU, at the national level, all other formulations with the same active ingredient, of which there can be hundreds [11], need individual approval. Which additional formulations trigger testing is determined by the similarity of their composition to already tested substances [21]. If their toxicity to bees can be predicted from existing data for formulations with a similar composition, then no additional testing is required. Formulations for which toxicity cannot be reliably predicted are not submitted to the full suite of ecotoxicological testing, but instead are benchmarked against existing products using mortality at a single dose to demonstrate equivalent toxicity [21].

Current regulatory regimes are insufficient to protect bees for three main reasons. First, the adjuvants that are added to these formulations via tank mixes undergo no bee toxicity testing at all [13,14], meaning that there is no regulatory data confirming their safety to bees (with the exclusion of limited testing in Germany [22]). An otherwise safe formulation could become toxic to bees if the adjuvant added is toxic [23]. Second, extensive data, including that collected by regulators, has demonstrated incredibly high variation in the toxicity of formulations with the same active ingredient to bees [24,25]. Finally, regulatory testing regimes are tailored to detect toxicity from potent insecticides capable of causing short-term mortality at low doses, not from ‘inert’ ingredients which may have more subtle, but still pertinent, sublethal effects at higher doses [26]. This could mean their toxicity is underestimated by regulatory testing.

Current understanding of the effects of ‘inert’ ingredients is almost exclusively centred around how they impact the toxicity of active ingredients [20,27]. Here, we focus on the individual impacts of ‘inert’ ingredients, rather than how they impact active ingredient toxicity, which is outside the scope of this review. It is important that we understand the effects of ‘inert’ ingredients in isolation because the ecological fate of each ingredient is unlikely to be uniform across the formulation [28,29].

Importantly, the development process of active ingredients makes them less likely to be ecologically persistent than ‘inert’ ingredients. Regulations, like maximum residue limits, that aim to cap consumer exposure incentivize agrochemical companies to produce active ingredients that readily degrade. There are no maximum residue limits for ‘inert’ ingredients [14], and as such no pressure to produce fast-decaying substances. For example, the pyrethroid insecticide deltamethrin has a half-life in pond water of less than 1 day [30]. By contrast, the surfactant adjuvant Multi-Film X-77, which can be applied as part of the same tank mix as pyrethroids, can repel honeybee visitation from a pond for six months after an initial spiking of 500 mg kg^−1^ [31,32]. This concentration of Multi-Film X-77 also causes honeybees to drown at high rates for 60 days after application [31]. In this scenario, the pyrethroid active ingredient has degraded well below the limit of detection while the ‘inert’ adjuvant is still causing significant mortality for months afterwards. This illustrates that assuming that all ingredients in a formulation will behave in a uniform manner once in the environment is unlikely to be true.

One of the reasons that there is a paucity of data on the environmental fate or toxicity of ‘inert’ ingredients’ is that, under EU law, only co-formulants with specific human hazard statements attached need to be reported as ingredients [33]. EU laws are nonetheless among the most stringent in the world, with comparable documents from the US having less information. The identity and concentration of other ingredients are explicitly protected under EU law as proprietary information [14]. Maintaining the identity of ‘inert’ ingredients as trade secrets severely impedes researchers' capacity to understand how they spread in, and affect, nature [25,34].

The limitations of current regulatory testing regimes are illustrated by the fate of the three neonicotinoid insecticides (imidacloprid, thiamethoxam and clothianidin) for which authorization for outdoor use was revoked in the EU in 2013 [19]. These substances had passed full bee ecotoxicological testing but were nonetheless later shown through academic research to cause serious detriment to bees and bee populations, as a result of sublethal effects that the regulatory process failed to detect [3,35]. Just as the limited scope of the regulatory system failed to detect the risk that these neonicotinoids posed to bees [36], ‘inert’ ingredients too could be damaging to bees without triggering concern during the regulatory process. Consequently, academic research has a significant role to play in assessing the exposure, hazards and risks associated with ‘inert’ ingredients.

Existing academic research on ‘inert’ ingredients has focussed on surfactants (most commonly as adjuvants) and solvents (most commonly as co-formulants). Surfactants (derived from surface active agent) are among the most common adjuvant types [12]. They function by reducing surface tension, enabling the spray to spread out over the surface of the leaf, increasing contact area and active ingredient uptake by the plant [37]. Solvents are co-formulants that allow an active ingredient to be dissolved at a higher concentration than if it were dissolved in water [7]. Because formulations are sold as concentrated stocks, this makes formulations cheaper to produce, distribute and store. Crop oil concentrates are a much less frequently studied type of ‘inert’ ingredient. They are typically petroleum-based spray adjuvants used to reduce droplet evaporation and aid degradation of the wax surface on a leaf, promoting active ingredient penetration. The substances described above are used widely in agriculture, and their impacts on bee health are not well understood. As such we use a systematic review approach to comprehensively summarize what is known about the effects of such ‘inert’ ingredients on bees.

## 2. Methods

Web of Science Core Collection and Google Scholar searches were undertaken based on the methods used by Cullen *et al*. [38] and Haddaway *et al*. [39], using the PRISMA framework [40], and combined with forward and backwards citation tracing to ensure that all relevant literature was captured. We acknowledge that using only the English language potentially excludes relevant literature. Full methods, including search terms, inclusion criteria and definitions are available in the electronic supplementary material.

The literature captured was not appropriate for a meta-analysis, so no quantitative analysis has been conducted. Peer-reviewed studies were included in the review if they presented experimental research testing at least one treatment of an agricultural co-formulant or adjuvant, with an appropriate control, or measured residues of an agricultural co-formulant or adjuvant in bees, honey, wax or bee-collected nectar or pollen. Because the word adjuvant is used to refer to co-formulants by some authors we define it here as meaning a separate product used as a tank additive [7].

## 3. Results

A total of 19 studies (from 1973 to 2021) fulfilled the inclusion criteria, comprising 16 experimental studies, two residue analysis studies, and one experimental and residue analysis study. There was a mixture of methodological approaches, with 12 laboratory, three semi-field and four field studies. However, diversity among study organisms was severely limited, with 16 studies testing honeybees, and just three studies on a species other than *Apis mellifera* (specifically, the bumblebee *Bombus terrestris*, and the solitary bees *Osmia lignaria* and *Megachile rotundata*). This demonstrates the lack of knowledge about how these widely applied substances could impact any of the other approximately 20 000 bee species [41].

Most studies (*n* = 14) tested surfactants, while some tested solvents (*n* = 4) and only one tested crop oil concentrates, stickers or wetting agents (*n* = 1). The life-history stage studied varied, with adults being the most commonly studied stage (*n* = 14), followed by larvae (*n* = 6), and then pupae (*n* = 2) and eggs (*n* = 1). Nearly all studies focused on mortality (*n* = 15), while food consumption was the second most studied metric (*n* = 5), followed by reproduction (*n* = 4). Among the studies measuring ‘inert’ ingredient residues, two focussed on surfactants, and one on solvents. In total, 56 substances or products have been experimentally tested in the academic literature, and just nine have been tested in more than one study, indicating a lack of depth of study for those tested. For further analysis of the studies included in this study, and the metrics extracted from them, see the electronic supplementary material. We note that seven of the post-2010 studies are from one network of authors. Further detail and a table summarizing the key findings of each study can be found in the electronic supplementary material.

The risk an agrochemical poses to bees is a combination of the exposure bees face and the likely consequences if exposed (hazard). Below, the research identified in this systematic review is divided into two sections: residue studies, which quantify exposure, and then experimental studies, which quantify hazard.

### (a) Residue studies

Because the ecological persistency of ‘inert’ ingredients in nature is poorly understood we do not know to what extent exposure occurs [28]. To address this question, it is possible to measure ‘inert’ ingredient residues in bee matrices, such as honey, pollen, nectar, wax and bees themselves. The limited evidence available has typically identified wax as a major substrate for residue accumulation [42,43]. Two studies have looked at various surfactants, and one at the solvent N-methyl-2-pyrrolidone (NMP).

Chen & Mullin [42] analysed trisiloxane surfactants in honeybee matrices. Trisiloxane surfactants are common surfactant co-formulants in the organosilicone group and are included in spray adjuvants like Silwet L-77 and Dyne-Amic. They can be used with a range of pesticide classes, and on a range of crops. Chen & Mullin [42] sampled honey, pollen and wax samples from seven US states, and while there were no positive detections in honey, 60% of pollen and all wax samples had positive detections (max. concentrations 39 µg kg^−1^ and 390 µg kg^−1^, respectively). The same authors later tested for nonylphenol ethoxylate and octylphenol ethoxylate surfactants in the same matrices [43]. Again, honey was the least contaminated (46 ± 26 µg kg^−1^, mean ± s.d.), followed by pollen (429 ± 203 µg kg^−1^) and wax (1051 ± 2897 µg kg^−1^). While Chen & Mullin [43] also identified ﻿trisiloxane surfactants residues in almond flowers, this study is not included in the systematic review results because the matrix analysed was not collected by bees. These studies demonstrate that bees are exposed to surfactants at non-negligible concentrations; however, whether these concentrations have a meaningful toxic impact is unknown, particularly as the experimental literature reviewed below typically uses much higher concentrations.

NMP is a solvent co-formulant often used in insecticide formulations [44]. Experimentally exposed honeybee larvae were less capable of metabolizing NMP residues than workers [44]. While another residue analysis study, Fine *et al*. [45] was excluded from this systematic review (because the matrices studied were not collected by bees), its results are still of interest as it is the only study in which an ‘inert’ ingredient was purposefully applied to a crop to enable the explicit measurement of residues in pollen or other bee relevant matrices [45]. Following the manufacturer's instructions, an insecticide formulation (Rimon 0.83EC), containing 40–50% NMP, was applied to apple trees either at the bud stage or while flowering. When sprayed at bud, a high of 22 000 µg kg^−1^ (17 150 ± 4390 µg kg^−1^) in pollen was detected 12 h after application, while direct application to the flowers found a high of 234 600 µg kg^−1^ in pollen 2.5 h after application [45]. These residue levels were 58 × higher than those of the active ingredient novaluron, demonstrating the high levels of exposure bees face.

The lack of exposure studies we identify here has important implications for experimental tests of hazard. For active ingredients, exposure regimes are typically designed with reference to the results of semi-field studies where the pesticide is deliberately applied to a crop [46]. Pollen and nectar brought back to the nest by foraging honeybees is collected and the pesticide levels quantified [47]. Using these data, chronic exposure scenarios can be constructed that assess the potential effects on individuals or colonies of bees foraging on a recently sprayed crop [46]. Without similar experiments for a range of ‘inert’ ingredients, it is not possible to inform experimental exposure regimes with real-world data.

### (b) Experimental studies

Given the general lack of exposure and residue studies we identify above, the only reference point we can use for exposure regimes in experimental studies is likely to be the in-tank mix concentration, which is the concentration of the ‘inert’ ingredient in the solution as sprayed. For co-formulants, this is not always known because their concentration and identity are not required to be publicly disclosed [14]. For adjuvants, most UK labels mandate a maximum concentration of 1% (1 part adjuvant to 99 parts formulation and water); somewhat crudely, a 1% solution equates to 10 000 mg kg^−1^ assuming equal densities. This means that without bioaccumulation we would expect around 10 000 mg kg^−1^ (1%) to be the very upper end of field-realistic exposure, which is equivalent to feeding directly on in-tank mix. While this may be appropriate for acute exposure (see [48]), it is likely to vastly overestimate field-realistic chronic exposure. The studies detailed below use a range of exposure levels that may or may not be field-realistic. Consequently, it is difficult to relate the toxicities observed to real-world risks. However, while little is known about the ecological persistencies of ‘inert’ ingredients’ and how they map to the ecotoxicological risk posed to bees, we have known for nearly a century that some surfactants can have strong insecticidal action with sufficient exposure.

Soaps, which are surfactants, have been recognized as posing risks to insects as far back as 1931 ([49], cited in [50]). The mechanism through which surfactants cause mortality in insects is unresolved, although Stevens [37] notes that insect spiracles are similar in size to plant stomata, which surfactants are designed to penetrate. Thus, surfactants may block the breathing apparatus of the insects and cause them to suffocate [25].

Adjuvants have been tested since the 1970s [31,32], and these studies found significant effects of surfactant adjuvants on honeybee drowning events when added to the bees' water supplies and repellence from the spiked water for up to six months. However, they found no evidence of deterrence from sprayed flowers, meaning that bees will not avoid contaminated flowers, and as such will be exposed to higher levels of surfactants. These types of studies have not been repeated since, meaning we do not know if the new generations of ‘inert’ ingredients cause similar effects.

Exposure to adjuvants is not limited to contamination of water sources, as farmers spray adjuvants in a range of situations, and labels do not include any guidance for reducing bees' exposure. As such, label guidance allows for direct overspray of bees, which could cause mortality through contact exposure. Contact exposure occurs when a bee is exposed to spray droplets of a pesticide, or when it lands on a recently sprayed surface such as a flower or leaf. In experimental studies, this is often simulated by either using a spraying apparatus to mimic direct overspray of bees, or by pipetting 2 µl of the pesticide onto the dorsal side of the thorax/abdomen of anaesthetized bees (OECD 214 [51]). Using a Potter spray tower, which replicates recommended spraying apparatus, two surfactant adjuvants, Pulse^®^ and Boost^®^, were found to cause 100% mortality in honeybees at 40–50% of the label-recommended concentration [52]. While the use of a Potter spray tower and label-recommended concentrations makes this study reasonably representative of in-field application, the application rate (l ha^−1^) used is likely an overestimate of realistic application; the rate of 2000 l ha^−1^ used for most experiments is an unrealistically high application rate in nearly all settings.

A recent study, Wernecke *et al*. [53], again used a Potter spray tower to apply surfactant adjuvants at field-realistic concentrations to anaesthetized honeybees, this time using a field-realistic application rate of 300 l ha^−1^. None of the six adjuvants tested caused mortality on their own. When paired with Goodwin & McBrydie, [52] which found considerable mortality, it is likely that at field-realistic concentrations some surfactant adjuvants cause mortality at an application rate between 300 l ha^−1^ and 2000 l ha^−1^. While the 300 l ha^−1^ used in Wernecke *et al*. [53] is field-realistic, it is not the worst-case exposure when following label guidelines. Several formulations of these adjuvants can be applied with have maximum application rates in the range of 800 l ha^−1^, meaning the amount of adjuvant could be up to 2.7 × higher. Further, if applied alongside a pesticide class like a herbicide, which have relatively high concentrations of surfactant [25], the overall amount of surfactant would be considerably higher, and thus more toxic. As such Wernecke *et al*. [53] does not rule out field-realistic toxicity of surfactant adjuvants, but does inform us that many adjuvant applications will not cause acute contact mortality.

Wernecke *et al*. [53] also found that when surfactant adjuvants, which did not cause mortality alone, were applied alongside insecticide formulations, which did not cause mortality alone, considerable mortality was observed. This indicates that surfactant adjuvants can meaningfully change the toxicity of insecticides from safe to toxic.

When testing surfactant toxicity, the methodology chosen is likely to influence the outcome. The standard contact toxicity test for honeybees, OECD 214 [51], has been used to determine the toxicity (hazard) of both Silwet L-77 and Triton X-100, with LD_50_'s of 357 µg bee^−1^ and 1436 µg bee^−1^, respectively [54]. This can be used to inform risk management strategies by allowing comparison of the toxicity with other substances. Donovan & Elliott, [55] used OECD 214 [51] to test the toxicity of several adjuvants, mostly surfactants, on honeybees and found no significant mortality from any substance. However, the dosing regime lacked the range needed to detect lethal effects and is insufficient to justify the conclusion that the substances tested were ‘non-toxic to honeybees'. This is because use of a 2 µl droplet applied to the thorax does not represent the degree of exposure bees can face in a field-realistic setting, which is poorly understood. As such the experiment under-exposed the bees by setting an artificial 2 µl limit while using field-realistic concentrations.

Chronic oral toxicity of surfactants has been tested on honeybees in two studies. Moffett & Morton [31] found two out of seven adjuvant/surfactant co-formulants caused mortality at the very high exposure level of 1000 mg kg^−1^ in nectar over 60 days (nearly equivalent to drinking in-tank mix for the entire honeybee worker lifespan). At 10 and 100 mg kg^−1^, no significant difference was detected from the control even over the full 60-day exposure period. By contrast, Chen, Fine & Mullin [34] suggested that three trisiloxane surfactants at 100 mg kg^−1^ reduced survival over an 8- or 10-day period. There was a clear effect of the class of surfactant, with trisiloxane surfactants causing greater than 90% mortality relative to the control, while alkylphenol polyethoxylates and fatty amine polyethoxylates surfactants caused less than 20%. These results indicate that the hazard surfactants pose could be mitigated by redesigning formulations/adjuvants to choose the safer options.

The effects of pesticides are not limited to mortality, and a vast body of research now documents the importance of sublethal impacts of agrochemicals for social bees [26,56]. For example, impairment of learning ability may impact upon foraging success [57], which may then impact colony reproductive success. Eleven studies have measured sublethal effects of ‘inert’ ingredients on bees, providing more information on their effects on fitness. Most notably, Ciarlo *et al*. [48] tested acute 20 µg bee^−1^ doses of several adjuvant products individually on honeybee learning using the proboscis extension reflex methodology. In the field, a honeybee feeding for just 2 s on sprayed tank mixture (which can be sprayed onto flowering crops or weeds) would imbibe a 20 µg dose of the surfactant adjuvants tested [48]. All 20 µg bee^−1^ doses of surfactant adjuvants impaired learning, but crop oil concentrates did not, suggesting that the different classes of ‘inert’ ingredient are toxicologically distinct.

Another important sublethal effect in social bees is queen rearing success, with reduced queen production being likely to reduce colony fitness. However, only two studies so far have examined this question. Johnson & Percel [58] found no effect of the surfactant adjuvant Break-Thru, at 200 mg kg^−1^ in *ad libitum* pollen, on several metrics of honeybee queen rearing success. In a follow-up study, Ricke, Lin & Johnson [59] fed bees *ad libitum* pollen spiked with the surfactant adjuvant Dyne-Amic at 0.8% by weight, which is likely an overestimate of realistic chronic exposure. No significant mortality was observed, and the adjuvant was not found to alter fungicide or insecticide active ingredient translocation from pollen to jelly [59]. It is notable that in managed colonies, for example, most honeybee colonies, queen rearing is less influential than in wild colonies due to the involvement of the beekeeper.

While the studies described above have looked at ‘inert’ ingredients individually, pressures on bee health are multifactorial [60–63], with novel stressors like agrochemicals adding to pre-existing stressors like parasites. Consequently, we may only be able to appreciate the impact pesticides have when we understand how they interact with other stressors. Only one study has tested the interaction between an ‘inert’ ingredient and a stressor other than another agrochemical. In a fully crossed experimental design, Fine *et al.* [64] spiked honeybee larval diets with 10 mg kg^−1^ of the surfactant adjuvant Sylgard 309 and a representative dose of a mixed virus inoculum. The surfactant adjuvant was found to increase black queen cell viral titre significantly, demonstrating an interaction between the stressors. Both stressors alone reduced larval survival, causing failed moults, melanization and other developmental abnormalities. When combined, the stressors acted synergistically, causing more larval mortality than the additive impacts of either stressors relative to the control.

Our systematic review revealed a severe lack of diversity in study organism, with only three studies testing ‘inert’ ingredients on bee species other than honeybees. Straw & Brown [26] fed individual bumblebees, *Bombus terrestris*, an acute dose of each co-formulant listed on the label of a widely used fungicide, Amistar, as well as the formulation as a whole. The experiment followed the regulatory guidelines for mortality testing (OECD 247 [65]), with the addition of the sublethal metrics, sucrose consumption, weight change and gut browning. Significant effects of the formulation were seen in all metrics, lethal and sublethal, but interestingly it was the surfactant/emulsifier co-formulant, alcohol ethoxylates, that was responsible for the whole toxicity of the formulation. The alcohol ethoxylates caused gut browning which indicates substantial damage to the gut. For the first time, this demonstrated that a co-formulant alone can drive the toxicity of a formulation.

In another experiment on a non-honeybee species, Ladurner *et al*. [66] tested the effects of the surfactant adjuvant Dyne-Amic on *Osmia lignaria* nesting behaviour and reproduction and reported no lethal or behavioural effects of Dyne-Amic. Artz & Pitts-Singer [67] tested the effects of the surfactant adjuvant N-90 on both *O. lignaria* and *Megachile rotunda* when sprayed on *Phacelia tanacetifolia* and *Sinapis alba* at label-recommended rates. In flight cages with the sprayed crops, nest recognition ability in both species was significantly impaired by N-90. While no mortality was found, these results are likely to be conservative, as the N-90 spray was applied at night when bees were not foraging, whereas label guidance for N-90 is unlikely to mandate night application, and so realistic field usage may result in direct contact with the spray, rather than residues that may have dried by the time bees become active.

Solvents are widely used co-formulants [68], yet only two solvents NMP and dimethyl sulfoxide (DMSO) have been tested on bees. These solvents are alternatives to one another with one producer of DMSO advertizing it as safer and less toxic than NMP [69].

All work on oral exposure to NMP has used a chronic feeding regime whereby NMP was administered in a sucrose solution, while the residue work has measured NMP in pollen, and as such it is difficult to assess the field realism of the exposure regimes in the experimental work.

As no residue analyses of field-realistic NMP nectar concentrations are currently available, a wide range of concentrations (0.537–10 000 mg kg^−1^) have been used in the exposure regimes in experimental work. The first study to assess NMP toxicity to honeybee larvae was Zhu *et al*. [68], which found 50% mortality within 12 h at 10 000 mg kg^−1^; however, in the absence of a control, these results cannot be interpreted (and as such this study is excluded from the systematic review results). When repeated, in a study by Fine *et al*. [45], 100 mg kg^−1^ of NMP caused significant larval mortality compared to the control, although mortality did not reach 50% over the 20-day trial period.

By contrast, adult honeybees only experienced significant mortality at concentrations as high as 5000 mg kg^−1^ [44], which is unlikely to be a field-realistic chronic exposure. This suggests that larvae are more susceptible to NMP than adults. The effects of chronic exposure to 500 mg kg^−1^ NMP for 7–10 days on honeybee colony health was also investigated by Fine *et al*. [45]. This concentration is above the 100 mg kg^−1^ that is known to cause larval mortality, but below the 5000 mg kg^−1^ that causes adult mortality. In this study, NMP inhibited colony weight gain and emerging forager counts, which is most likely to be caused by larval mortality and knock-on effects on colony foraging.

To investigate whether higher impacts of NMP on larvae were a function of differential detoxification, Fine & Mullin [44] fed honeybee workers and larvae 200 mg kg^−1^ of NMP in nectar for 6 days and quantified residues of the NMP and its metabolites from the adults and larvae. They found that larvae were less able to detoxify the NMP, and this may explain the higher sensitivity of larvae to NMP. Using OECD 214, NMP was found to have an acute contact LD_50_ greater than 2000 µg bee^−1^ [51,54]. This finding suggests NMP is of negligible toxicity when applied via acute contact.

DMSO has received less attention than NMP, with only two studies assessing its toxicity to bees. Moffett & Morton [31] found that DMSO produced no significant lethal effects in honeybees with chronic exposure of 1000 mg kg^−1^ for 60 days. Milchreit *et al*. [70] found mixed effects of chronic oral exposure (500 mg kg^−1^) on honeybee brood development, with no detriment to fitness clearly demonstrated. Together, these results support the producer's assertion that this substance is less toxic than its alternative NMP [69].

## 4. Discussion

### (a) A call to reprioritize research into ‘inert’ ingredients

Research into the effects of pesticides on bees is disproportionately focussed on active ingredients, with ‘inert’ ingredients receiving significantly less attention. This is most clearly visible when considering the number of studies focussing on them relative to the best-studied pesticide class, insecticides. For example, a single active ingredient, the neonicotinoid imidacloprid, was the subject of 168 studies as of 2015 [71]. This dwarfs the literature on ‘inert’ ingredients, with the systematic review here finding just 19 studies up to 2021. The allocation of research is partially explained by the intended purpose of insecticides—to kill insects. However, as we detail above, despite ‘inert’ ingredients not being designed to kill insects, they can have unintended consequences on bee health.

If bee ecotoxicological research is an applied science with the aim of understanding the risks pesticides could pose to bees, the optimal allocation of research effort to substances should match the potential risk each substance poses. This risk is a combination of the hazard posed to bees and the likelihood of exposure. The hazard is likely greatest with insecticides. However, exposure is likely to be greatest with ‘inert’ ingredients that are used in far higher quantities [20], with little in the way of exposure mitigation. The current allocation of research effort has focussed strongly on the hazard posed by insecticides, without recognizing that ‘inert’ ingredients have vastly higher exposure levels. This means that the allocation of research is primarily based on hazard, not risk as it should be (table 1). As such, research effort should be reallocated to inert ingredients to characterize their exposure and hazard to bees, after which the benefits of further research can be evaluated.

**Table 1.** Detailing the hazard, exposure and risk insecticides and ‘inert’ ingredients pose to bees. Risk = hazard * exposure.

	hazard	exposure	risk
insecticide	high	low-stringent mitigation measures	intermediate
‘inert’ ingredients	poorly characterized but non-negligible	very high-little to no mitigation measures	intermediate, but poorly characterized

To be clear, research into insecticidal active ingredients is in our opinion clearly justified, but a reallocation of resources to better reflect the risks bees face in the wild would encompass ‘inert’ ingredients as well. Applied bee pesticide research would therefore benefit from allocating resources to agrochemicals in proportion to their potential risk to bees. This would require research into large numbers of chemicals that may have never been tested on bees before. We propose that the potential, and likely impacts of these widely applied substances on bee health represents a key knowledge gap that urgently requires research attention and funding.

While we contend that exposure will be high for ‘inert’ ingredients, the data to support this statement are severely limited. If regulatory bodies were to mandate residue analysis for all agrochemicals, including ‘inert’ ingredients, we would have a better understanding of the complex exposure bees face. A well-funded and systematic approach to residue monitoring required is something only a regulatorily mandated process can offer. Without this, academic researchers will not be able to properly assess whether their exposure regimes are field-realistic, which could lead to unsubstantiated estimates of the risks that ‘inert’ ingredients pose to bees.

## 5. Conclusion

The literature reviewed above raises a number of concerns around the impacts of ‘inert’ ingredients on bee health and productivity at the individual and colony levels. What little research we have on ‘inert’ ingredient residues in nature shows them to be widespread, and at high concentrations [42,43,45], although our understanding of what the concentration range of ‘inert’ ingredients is in agricultural systems is underdeveloped. Importantly, and in addition to this limited understanding of environmental residues, the research identified here demonstrates that ‘inert’ ingredients are not ecotoxicologically benign, and as such they should be subject to greater regulation.

‘Inert’ ingredients drive mortality through multiple exposure routes [26,31,32,52], synergize with other stressors [53,64] and cause sublethal effects [26,48,67]. While we call on regulators to require testing of ‘inert’ ingredients on bees, we also caution that the current regulatory testing system is ill-equipped to test the effects of ‘inert’ ingredients. Current regulatory testing exclusively uses methodologies designed for neurotoxic insecticides, which may not properly characterize the risks of ‘inert’ ingredients that are less potent, but have higher exposure levels [26]. Given that surfactants have been identified as causing both sublethal [26,48,67] and synergistic effects alongside other stressors [53,64], a regulatory testing approach that measures sublethal effects and incorporates multiple stressors is essential.

‘Inert’ ingredients interact with a range of stressors, but perhaps most importantly with active ingredients. A systematic comparison of active ingredient toxicity versus whole formulation toxicity covering academic and regulatory data would give highly informative results, but is outside of the scope of this review. As prior reviews have demonstrated [20,27], formulations are commonly more toxic to non-target organisms than active ingredients, suggesting that the term ‘inert’ ingredients may not be appropriate. In fact, the use of the words ‘inert’ or ‘inactive’ to describe co-formulants and adjuvants posits that they are toxicologically benign substances. The research collated here demonstrates that this is not true for all such substances and highlights a lack of data for many more. There is, however, currently too little evidence to make broad conclusions about ‘inert’ ingredients in general, or for any individual bee species. As such we would suggest that the terms ‘co-formulant’ or ‘adjuvant’, where appropriate, are better descriptors of the substances because they are neutral regarding their toxicological activity.

Just as the language used to describe ‘inert’ ingredients does not reflect their potential toxicity, neither does the legislation regulating them. Legislation that protects formulation composition as trade secrets hampers research into the impacts of ‘inert’ ingredients [10,14,20,25], as such publication of formulation composition would be a critical step forward for environmental risk assessment.

Progress has come with the recent European Commission legislation on co-formulants [72], where the ostensible aim is to ban co-formulants harmful to humans or the environment. However, the legislation will only effect change if the European regulatory process is adapted accordingly. EFSA have made progress in this area with proposals to regulate by product, and with explicit consideration of the co-formulants [73], but this has yet to become practice. The progress in regulating co-formulants has almost exclusively been driven by human toxicity concerns, with little consideration given to other non-target organisms [74]. Despite these proposals for co-formulants, adjuvants are still entirely unregulated at the European level, despite many containing the same chemicals as many co-formulants [72]. Nationally, the only progress has come from the Germany pesticide regulation authority, the BVL. The BVL reacted to Wernecke *et al*. [53], which found surfactant adjuvants interact with insecticides to cause mortality, by requiring limited contact testing of adjuvants alone and alongside some insecticides [22]. This is the first legislation, to our knowledge, to explicitly require bee toxicity testing for an adjuvant.

In conclusion, evidence of ‘inert’ ingredients having the potential to cause mortality in bees dates back to the 1970s [31], yet in the EU and US, there is still no regulatorily mandated toxicity testing of ‘inert’ ingredients [14]. This means that the only currently available research stream is academic testing, which has produced just 19 studies to date. This represents a large gap in our understanding of pesticide ecotoxicology. The research collated here demonstrates that ‘inert’ ingredients are not inert and can pose significant risks to bee health. We call on researchers to devote more attention to ‘inert’ ingredients and regulators to require testing of ‘inert’ ingredients to ensure their safety to bees.

## Data Availability

The data extracted from the systematic review literature are available in the electronic supplementary material [75].
